# Supplementary material for: Sperm physiology and in vitro fertilising ability rely on basal metabolic activity: insights from the pig model
Source: Commun Biol. 2023 Mar 30;6:344. doi: 10.1038/s42003-023-04715-3 (PMC10063579; doi:10.1038/s42003-023-04715-3)
Supplement: Supplementary file 2 — Supplementary Information [file 42003_2023_4715_MOESM2_ESM.pdf]

Supplementary information

Supplementary Figure 1. Gating strategy for (**a**) viability, (**b**) acrosome integrity, (**c**) intracellular calcium levels, and (**d**) mitochondrial membrane potential.

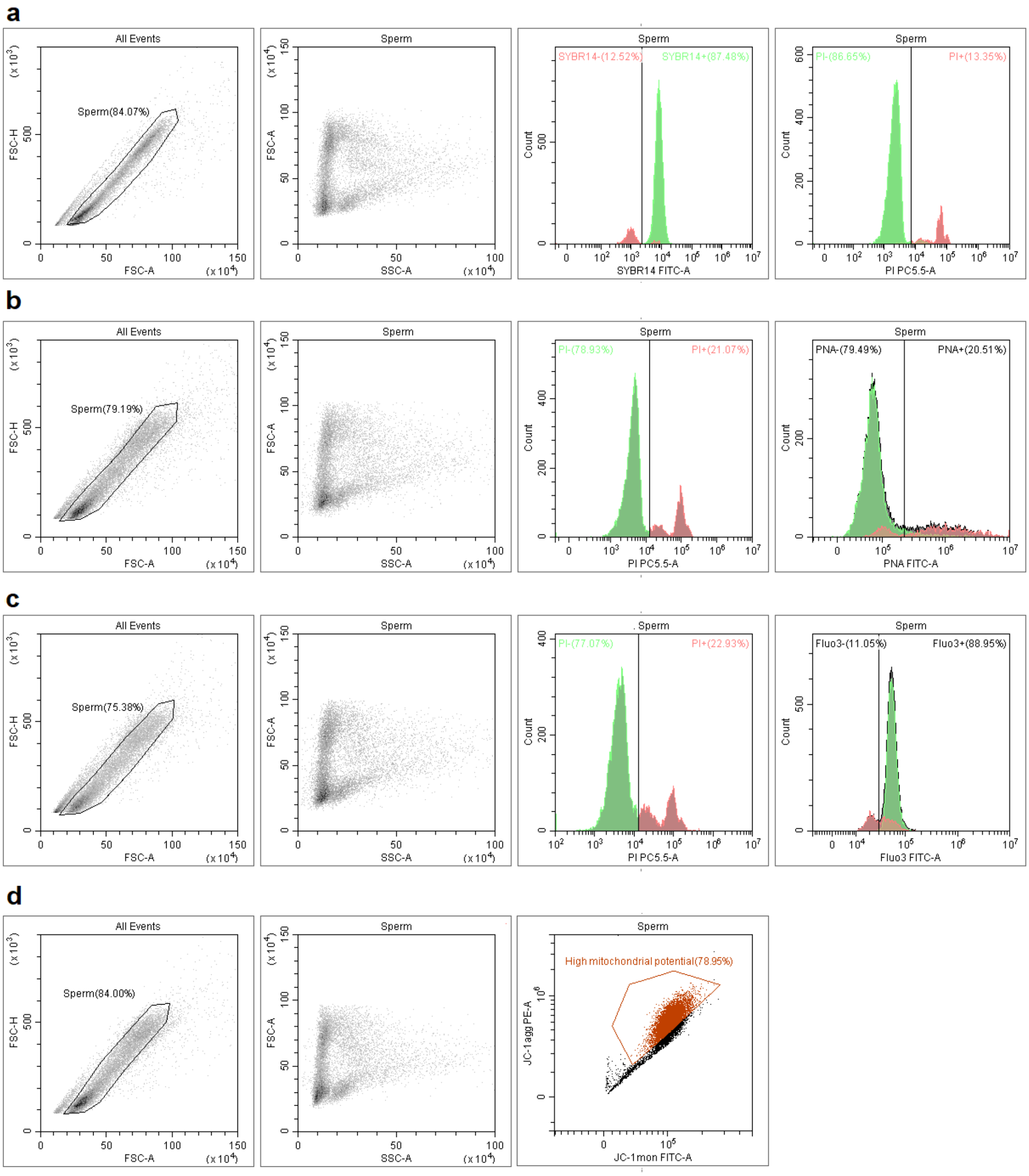

Supplementary Table 1. Variable description.

Feature	Block	Mean ± SD	Loading value[a]
Viable sperm (%)	Sperm Quality	90.289 ± 3.598	0.469
Progressive Motility (%)	Sperm Quality	72.249 ± 10.362	0.488
Total Motility (%)	Sperm Quality	88.467 ± 5.386	0.519
Sperm with normal morphology (%)	Sperm Quality	92.880 ± 7.856	0.522
Intracellular calcium levels (AU)	Sperm Function	36385.28 ± 43320.62	-0.703
Sperm with high mitochondrial membrane potential (%)	Sperm Function	85.661 ± 6.780	-0.617
Viable sperm with an intact acrosome (%)	Sperm Function	84.973 ± 3.943	-0.355
Fertilisation Rate (%)	IVF Outcomes	37.030 ± 11.802	0.211
Developmental Ratio	IVF Outcomes	2.354 ± 2.474	0.253
Developmental competency of fertilised oocytes	IVF Outcomes	1.408 ± 0.47	0.307
Morulae (%)	IVF Outcomes	12.798 ± 6.704	0.325
Hatching/Hatched blastocysts (%)	IVF Outcomes	0.963 ± 1.772	0.359
Early blastocysts/blastocysts (%)	IVF Outcomes	13.570 ± 7.920	0.367
Total number of embryos (%)	IVF Outcomes	45.186 ± 12.128	0.405
Morulae and total blastocysts (%)	IVF Outcomes	27.329 ± 11.16	0.513

[a]Loading values in the projection to the first principal component from principal component analysis. Abbreviations: IVF, in vitro fertilisation. PC: principal component. AU: arbitrary units.

Supplementary Table 2. Correlation between components from the multi-block data integration*.

	Component 1 Sperm Quality	Component 2 Sperm Quality	Component 1 Sperm Function	Component 2 Sperm Function	Component 1 IVF Outcomes	Component 2 IVF Outcomes
Component 1 metabolomics	0.73	0.14	0.96	0.01	0.78	0.11
Component 2 metabolomics	-0.51	0.52	0.08	0.89	0.08	0.50
Component 1 Sperm Quality	-	-	0.65	-0.37	0.53	-0.09
Component 2 Sperm Quality	-	-	0.03	0.75	0.30	0.71
Component 1 Sperm Function	-	-	-	-	0.78	0.14
Component 2 Sperm Function	-	-	-	-	0.03	0.52

* Components 1 and 2 corresponded to the latent components of the N-multiblock data analysis..

Supplementary Table 3. Unfolded pair-wise similarity scores from the multi-block data integration.

Feature block X	Feature block Y	Similarity Score
Intracellular calcium levels	Morulae and total blastocysts	-0.98
Progressive Motility	Intracellular calcium levels	-0.90
Citrate	Intracellular calcium levels	-0.90
Sperm with normal morphology	Intracellular calcium levels	-0.89
Citrate/Lactate	Intracellular calcium levels	-0.88
Isocitrate/Citrate	Sperm with high mitochondrial membrane potential	-0.87
Citrate/Malate	Intracellular calcium levels	-0.87
Intracellular calcium levels	Total embryos	-0.87
Isocitrate/Citrate	Fertilisation Rate	-0.85
α-Ketoglutarate	Viable sperm with an intact acrosome	-0.81
Intracellular calcium levels	Hatching/Hatched blastocysts	-0.80
Isocitrate/Citrate	Viable sperm with an intact acrosome	-0.77
α-OH-glutarate/Isocitrate	Morulae	-0.76
Isocitrate/Citrate	Early blastocysts/blastocysts	-0.76
α-OH-glutarate/Isocitrate	Developmental competency of fertilised oocytes	-0.76
Fumarate/Succinate	Fertilisation Rate	-0.76
Fumarate/Succinate	Early blastocysts/blastocysts	-0.76
Isocitrate	Viable sperm with an intact acrosome	-0.73
α-Ketoglutarate/Isocitrate	Morulae and total blastocysts	-0.73
Fumarate/Succinate	Sperm with high mitochondrial membrane potential	-0.72
α-Ketoglutarate	Sperm with high mitochondrial membrane potential	-0.71
Intracellular calcium levels	Morulae	-0.71
Viable sperm	Intracellular calcium levels	-0.69
α-Ketoglutarate/Isocitrate	Sperm with normal morphology	-0.67
α-Ketoglutarate/Isocitrate	Progressive Motility	-0.67
Acetoacetate	Sperm with high mitochondrial membrane potential	-0.67
Isocitrate	Intracellular calcium levels	-0.66
Viable sperm with an intact acrosome	Developmental competency of fertilised oocytes	-0.66
α-Ketoglutarate/Isocitrate	Total embryos	-0.66
Acetoacetate	Viable sperm with an intact acrosome	-0.65
Intracellular calcium levels	Early blastocysts/blastocysts	-0.65
α-OH-glutarate/α-Ketoglutarate	Developmental competency of fertilised oocytes	-0.65
Intracellular calcium levels	Developmental competency of fertilised oocytes	-0.63
α-OH-glutarate/Isocitrate	Morulae and total blastocysts	-0.63

α-Ketoglutarate/Isocitrate	Morulae	-0.63
α-OH-glutarate/Isocitrate	Total embryos	-0.61
α-Ketoglutarate/Isocitrate	Hatching/Hatched blastocysts	-0.61
α-OH-glutarate/Isocitrate	Sperm with normal morphology	-0.61
α-OH-glutarate/α-Ketoglutarate	Morulae	-0.61
Malate	Viable sperm with an intact acrosome	-0.59
Isocitrate	Sperm with high mitochondrial membrane potential	-0.59
Acetoacetate	Fertilisation Rate	-0.59
Fumarate/Succinate	Viable sperm with an intact acrosome	-0.59
Malate	Sperm with high mitochondrial membrane potential	-0.59
α-OH-glutarate/Isocitrate	Progressive Motility	-0.58
α-Ketoglutarate/Isocitrate	Developmental competency of fertilised oocytes	-0.58
Viable sperm with an intact acrosome	Morulae	-0.57
Fumarate	Viable sperm with intact acrosome	-0.56
α-OH-glutarate/Isocitrate	Hatching/Hatched blastocysts	-0.56
Fumarate/Succinate	Morulae and total blastocysts	-0.55
Intracellular calcium levels	Developmental ratio	-0.54
Fumarate	Sperm with high mitochondrial membrane potential	-0.53
Sperm with high mitochondrial membrane potential	Developmental competency of fertilised oocytes	-0.53
α-Ketoglutarate	Intracellular calcium levels	-0.52
Citrate	Viable sperm with an intact acrosome	-0.52
Fumarate/Succinate	Progressive Motility	-0.51
α-Ketoglutarate/Isocitrate	Viable sperm	-0.51
α-Ketoglutarate	Fertilisation Rate	-0.50
Malate	Fertilisation Rate	-0.49
Fumarate/Succinate	Sperm with normal morphology	-0.46
Succinate	Intracellular calcium levels	-0.45
Acetoacetate	Early blastocysts/blastocysts	-0.44
Isocitrate/Citrate	Morulae and total blastocysts	-0.44
α-OH-glutarate/Isocitrate	Viable sperm	-0.44
Fumarate/Succinate	Total embryos	-0.42
Sperm with high mitochondrial membrane potential	Morulae	-0.42
Fumarate	Fertilisation Rate	-0.41
Isocitrate/Citrate	Progressive Motility	-0.41
Fumarate/Succinate	Viable sperm	-0.40
α-Ketoglutarate/Isocitrate	Early blastocysts/blastocysts	-0.40
Fumarate/Succinate	Developmental ratio	-0.39
α-Ketoglutarate/Isocitrate	Developmental ratio	-0.38
Fumarate/Succinate	Hatching/Hatched blastocysts	-0.38

Lactate	Morulae and total blastocysts	-0.38
Isocitrate/Citrate	Sperm with normal morphology	-0.35
Lactate	Progressive Motility	-0.35
Isocitrate	Fertilisation Rate	-0.35
Isocitrate/Citrate	Developmental ratio	-0.34
Succinate/α-Ketoglutarate	Developmental competency of fertilised oocytes	-0.34
Lactate	Sperm with normal morphology	-0.34
Isocitrate/Citrate	Viable sperm	-0.33
Malate	Early blastocysts/blastocysts	-0.32
Lactate	Total embryos	-0.32
Citrate/Lactate	Viable sperm with an intact acrosome	-0.31
Citrate	Sperm with high mitochondrial membrane potential	-0.31
Lactate	Early blastocysts/blastocysts	-0.31
Isocitrate/Citrate	Total embryos	-0.30
α-Ketoglutarate	Hatching/Hatched blastocysts	0.31
α-Ketoglutarate	Sperm with normal morphology	0.32
Viable sperm	Developmental competency of fertilised oocytes	0.32
Isocitrate	Viable sperm	0.32
Acetoacetate	Morulae	0.33
α-OH-glutarate	Sperm with normal morphology	0.33
Succinate	Viable sperm	0.33
α-Ketoglutarate	Total embryos	0.34
Isocitrate/Citrate	Developmental competency of fertilised oocytes	0.34
Fumarate/Succinate	Intracellular calcium levels	0.35
Lactate	Intracellular calcium levels	0.35
Malate	Morulae	0.35
α-Ketoglutarate/Isocitrate	Viable sperm with an intact acrosome	0.36
Succinate	Early blastocysts/blastocysts	0.37
α-OH-glutarate	Progressive Motility	0.37
Citrate	Early blastocysts/blastocysts	0.37
Succinate	Hatching/Hatched blastocysts	0.37
Succinate/α-Ketoglutarate	Early blastocysts/blastocysts	0.39
Fumarate	Morulae	0.39
Viable sperm	Morulae	0.40
α-OH-glutarate	Morulae and total blastocysts	0.40
Acetoacetate	Developmental competency of fertilised oocytes	0.40
Viable sperm	Fertilisation Rate	0.41
Succinate	Total embryos	0.41
Citrate	Developmental ratio	0.41
Malate	Developmental competency of fertilised oocytes	0.41
Succinate	Sperm with normal morphology	0.42

Progressive Motility	Developmental competency of fertilised oocytes	0.43
Fumarate	Developmental competency of fertilised oocytes	0.43
Viable sperm	Developmental ratio	0.44
Succinate	Progressive Motility	0.44
Isocitrate	Progressive Motility	0.44
Isocitrate	Hatching/Hatched blastocysts	0.44
Sperm with normal morphology	Fertilisation Rate	0.45
α-OH-glutarate/α-Ketoglutarate	Intracellular calcium levels	0.45
Citrate/Lactate	Developmental ratio	0.45
Sperm with normal morphology	Developmental competency of fertilised oocytes	0.45
Citrate/Malate	Developmental ratio	0.46
Isocitrate	Sperm with normal morphology	0.47
Succinate	Morulae and total blastocysts	0.47
Isocitrate	Morulae and total blastocysts	0.47
α-OH-glutarate	Viable sperm with an intact acrosome	0.47
Isocitrate	Total embryos	0.48
α-OH-glutarate/Isocitrate	Sperm with high mitochondrial membrane potential	0.50
Progressive Motility	Fertilisation Rate	0.51
Citrate/Lactate	Early blastocysts/blastocysts	0.51
α-OH-glutarate/α-Ketoglutarate	Fertilisation Rate	0.51
Succinate/α-Ketoglutarate	Fertilisation Rate	0.51
Viable sperm with an intact acrosome	Early blastocysts/blastocysts	0.52
Progressive Motility	Morulae	0.53
Citrate/Malate	Early blastocysts/blastocysts	0.54
Sperm with normal morphology	Developmental ratio	0.54
Sperm with normal morphology	Morulae	0.55
Citrate	Viable sperm	0.56
Succinate/α-Ketoglutarate	Viable sperm with an intact acrosome	0.56
Progressive Motility	Developmental ratio	0.57
α-OH-glutarate	Sperm with high mitochondrial membrane potential	0.57
Citrate/Malate	Developmental competency of fertilised oocytes	0.57
α-OH-glutarate	Early blastocysts/blastocysts	0.57
Succinate/α-Ketoglutarate	Sperm with high mitochondrial membrane potential	0.58
Viable sperm	Hatching/Hatched blastocyst	0.58
Citrate/Lactate	Viable sperm	0.59
Citrate/Malate	Viable sperm	0.59
α-OH-glutarate	Fertilisation Rate	0.59
Citrate/Lactate	Developmental competency of fertilised oocytes	0.60
Viable sperm	Early blastocysts/blastocysts	0.62

Citrate/Malate	Morulae	0.63
Viable sperm	Total embryos	0.63
Citrate/Lactate	Morulae	0.66
α-Ketoglutarate	Morulae	0.66
α-OH-glutarate/Isocitrate	Viable sperm with an intact acrosome	0.68
Citrate	Hatching/Hatched blastocysts	0.68
Citrate/Malate	Hatching/Hatched blastocysts	0.69
Citrate/Lactate	Hatching/Hatched blastocysts	0.69
α-Ketoglutarate	Developmental competency of fertilised oocytes	0.70
α-OH-glutarate/α-Ketoglutarate	Sperm with high mitochondrial membrane potential	0.71
Isocitrate	Morulae	0.71
Sperm with high mitochondrial membrane potential	Early blastocysts/blastocysts	0.73
Isocitrate	Developmental competency of fertilised oocytes	0.73
Citrate	Developmental competency of fertilised oocytes	0.73
Citrate	Total embryos	0.73
Citrate	Progressive Motility	0.74
Sperm with normal morphology	Early blastocysts/blastocysts	0.74
Viable sperm	Morulae and total blastocysts	0.74
Sperm with normal morphology	Hatching/Hatched blastocysts	0.74
Citrate	Sperm with normal morphology	0.74
Citrate/Malate	Total embryos	0.75
Citrate/Lactate	Total embryos	0.75
Progressive Motility	Hatching/Hatched blastocysts	0.76
Citrate	Morulae	0.76
Citrate/Lactate	Sperm with normal morphology	0.77
Citrate/Malate	Sperm with normal morphology	0.77
Citrate/Lactate	Progressive Motility	0.77
Citrate/Malate	Progressive Motility	0.77
α-OH-glutarate/α-Ketoglutarate	Viable sperm with an intact acrosome	0.78
Viable sperm with an intact acrosome	Fertilisation Rate	0.79
α-Ketoglutarate/Isocitrate	Intracellular calcium levels	0.79
Progressive Motility	Early blastocysts/blastocysts	0.79
Citrate	Morulae and total blastocysts	0.79
α-OH-glutarate/Isocitrate	Intracellular calcium levels	0.80
Sperm with normal morphology	Total embryos	0.81
Progressive Motility	Total embryos	0.83
Citrate/Lactate	Morulae and total blastocysts	0.83
Citrate/Malate	Morulae and total blastocysts	0.83
Sperm with high mitochondrial membrane potential	Fertilisation Rate	0.91
Sperm with normal morphology	Morulae and total blastocysts	0.93

Viable sperm with an intact acrosome	Sperm with high mitochondrial membrane potential	0.95
Progressive Motility	Morulae and total blastocysts	0.96

The network represented in Figure 2 was built using the unfolded pair-wise similarity matrix.

Similarity scores can be interpreted as correlation factors.
