# Supplementary material for: Sperm physiology and in vitro fertilising ability rely on basal metabolic activity: insights from the pig model
Source: Commun Biol. 2023 Mar 30;6:344. doi: 10.1038/s42003-023-04715-3 (PMC10063579; doi:10.1038/s42003-023-04715-3)
Supplement: Supplementary file 3 — Description of Additional Supplementary Files [file 42003_2023_4715_MOESM3_ESM.pdf]

Description of Additional Supplementary Files

File name: Supplementary Data

Description: The datasets used and/or analysed during this study
