# Supplementary material for: Sperm physiology and in vitro fertilising ability rely on basal metabolic activity: insights from the pig model
Source: Commun Biol. 2023 Mar 30;6:344. doi: 10.1038/s42003-023-04715-3 (PMC10063579; doi:10.1038/s42003-023-04715-3)
Supplement: Supplementary file 5 — Reporting Summary [file 42003_2023_4715_MOESM5_ESM.pdf]

nature portfolio

Corresponding author(s): Oscar J. Pozo
Marc Yeste

Last updated by author(s): Mar 9, 2023

Reporting Summary

Nature Portfolio wishes to improve the reproducibility of the work that we publish. This form provides structure for consistency and transparency in reporting. For further information on Nature Portfolio policies, see our Editorial Policies and the Editorial Policy Checklist.

Statistics

For all statistical analyses, confirm that the following items are present in the figure legend, table legend, main text, or Methods section.

n/a	Confirmed	
☐	☒	The exact sample size (*n*) for each experimental group/condition, given as a discrete number and unit of measurement
☐	☒	A statement on whether measurements were taken from distinct samples or whether the same sample was measured repeatedly
☐	☒	The statistical test(s) used AND whether they are one- or two-sided<br>*Only common tests should be described solely by name; describe more complex techniques in the Methods section.*
☐	☒	A description of all covariates tested
☐	☒	A description of any assumptions or corrections, such as tests of normality and adjustment for multiple comparisons
☐	☒	A full description of the statistical parameters including central tendency (e.g. means) or other basic estimates (e.g. regression coefficient) AND variation (e.g. standard deviation) or associated estimates of uncertainty (e.g. confidence intervals)
☐	☒	For null hypothesis testing, the test statistic (e.g. *F*, *t*, *r*) with confidence intervals, effect sizes, degrees of freedom and *P* value noted<br>*Give P values as exact values whenever suitable.*
☒	☐	For Bayesian analysis, information on the choice of priors and Markov chain Monte Carlo settings
☐	☒	For hierarchical and complex designs, identification of the appropriate level for tests and full reporting of outcomes
☐	☒	Estimates of effect sizes (e.g. Cohen's *d*, Pearson's *r*), indicating how they were calculated

Our web collection on statistics for biologists contains articles on many of the points above.

Software and code

Policy information about availability of computer code

Data collection	Commercial software was used to assess sperm motility (ISAS software; Integrated Sperm Analysis System, ISAS V1.0; Proiser S.L.; Valencia, Spain) and analyse flow cytometry results (CytExpert; Beckman Coulter Inc, 2.5.0.77; California, USA).<br>In addition, metabolome profile was acquired utilizing the software Software MassLynx - Waters Corporation.
Data analysis	Data preprocessing and statistical analyses were conducted using the open-source R software version 4.2.0.

For manuscripts utilizing custom algorithms or software that are central to the research but not yet described in published literature, software must be made available to editors and reviewers. We strongly encourage code deposition in a community repository (e.g. GitHub). See the Nature Portfolio guidelines for submitting code & software for further information.

Data

Policy information about availability of data

All manuscripts must include a data availability statement. This statement should provide the following information, where applicable:

- Accession codes, unique identifiers, or web links for publicly available datasets
- A description of any restrictions on data availability
- For clinical datasets or third party data, please ensure that the statement adheres to our policy

The datasets used and/or analysed during the current study are available in Supplementary Information.

Human research participants

Policy information about studies involving human research participants and Sex and Gender in Research.

Reporting on sex and gender	NA
Population characteristics	NA
Recruitment	NA
Ethics oversight	NA

Note that full information on the approval of the study protocol must also be provided in the manuscript.

Field-specific reporting

Please select the one below that is the best fit for your research. If you are not sure, read the appropriate sections before making your selection.

☒ Life sciences ☐ Behavioural & social sciences ☐ Ecological, evolutionary & environmental sciences

For a reference copy of the document with all sections, see nature.com/documents/nr-reporting-summary-flat.pdf

Life sciences study design

All studies must disclose on these points even when the disclosure is negative.

Sample size	We utilized the “pwr.f2.test” function from the “pwr” R pacakage to determine the minimum sample size considering the conditions of linear regressions. Considering an effect size of a 35 % (parameter “f2” in formula) based on previous metabolomics studies (i.e. 10.1016/j.jpba.2021.114450, 10.3390/ijms23063219), an alpha error of 0.05 (“sig.level” in formula) minimum power of the test of 60 % we estimated a minimum degree of freedom of 14.13 (equivalent to 14.13 + 1 = 15.13 observations). Therefore, we analysed 16 samples which is a suitable to produce robust results (explained in line 461). In addition, we want to note that we included a similar sample size compared to previous works (i.e. 10.3390/biom10060906, 10.1016/j.theriogenology.2022.12.009, 10.1016/j.jprot.2022.104791, 10.1111/rda.14270, 10.1002/mrd.23354, 10.1071/RD20304.).
Data exclusions	Data from all animals were included.
Replication	Data were analysed in a repeated-double cross-validation frame in which the population was divided into three sets: i) a training set; ii) a validation set for the construction of the statistical models; and iii) a set for an additional validation (two levels of validation) on a part of the population that has not been involved in either the training of the models or their external validation (see DOI: 10.1093/bioinformatics/bty710).
Randomization	No experimental groups were included in the present work.
Blinding	Metabolomic analysis and the subsequent statistical analysis were conducted blinded from each animal in vitro fertility results.

Reporting for specific materials, systems and methods

We require information from authors about some types of materials, experimental systems and methods used in many studies. Here, indicate whether each material, system or method listed is relevant to your study. If you are not sure if a list item applies to your research, read the appropriate section before selecting a response.

Materials & experimental systems

n/a	Involved in the study	
☒	☐	Antibodies
☒	☐	Eukaryotic cell lines
☒	☐	Palaeontology and archaeology
☐	☒	Animals and other organisms
☒	☐	Clinical data
☒	☐	Dual use research of concern

Methods

n/a	Involved in the study	
☒	☐	ChIP-seq
☐	☒	Flow cytometry
☒	☐	MRI-based neuroimaging

Animals and other research organisms

Policy information about studies involving animals; ARRIVE guidelines recommended for reporting animal research, and Sex and Gender in Research

Laboratory animals	NA
Wild animals	NA
Reporting on sex	Considering that this work aimed to determine sperm metabolism effect on embryo development, the sex of the animals used in each type of samples is implicit.
Field-collected samples	Semen samples were provided by a local artificial insemination centre (Gepork S.L.; Masies de Roda, Spain), which follows the ISO certification (ISO-9001:2008) and the EU Directive 2010/63/EU for animal experiments, the Animal Welfare Law issued by the Regional Government of Catalonia, and the current regulation on Health and Biosafety issued by the Department of Agriculture, Livestock, Food and Fisheries, Regional Government of Catalonia, Spain. On the other hand, ovaries were recovered from pre-pubertal gilts sacrificed for food purposes at a local abattoir (Frigorífics Costa Brava; Riudellots de la Selva, Girona).
Ethics oversight	As ejaculates were commercially acquired from an artificial insemination centre and animals were not manipulated for the sole purpose of the present experiment, permission from an Ethics Committee was not required.

Note that full information on the approval of the study protocol must also be provided in the manuscript.

Flow Cytometry

Plots

Confirm that:

☒ The axis labels state the marker and fluorochrome used (e.g. CD4-FITC).

☒ The axis scales are clearly visible. Include numbers along axes only for bottom left plot of group (a 'group' is an analysis of identical markers).

☒ All plots are contour plots with outliers or pseudocolor plots.

☒ A numerical value for number of cells or percentage (with statistics) is provided.

Methodology

Sample preparation	Sperm viability was assessed following the protocol of Garner and Johnson50, which uses SYBR-14, that stains sperm nuclei, and propidium iodide (PI), that only stains sperm having a compromised plasma membrane integrity. Briefly, semen samples were adjusted to a final concentration of 4×106 sperm/mL in 1× phosphate buffered saline (PBS). Next, samples were incubated for 15 min at 38 ºC with SYBR-14 (final concentration: 32 nM) and PI (final concentration: 7.5 µM). Stained cells were analysed using a CytoFLEX cytometer (Beckman Coulter; Fullerton, CA, USA), where SYBR-14 fluorescence was detected by the fluorescein isothiocyanate (FITC) channel (525/40), and PI using the PC5.5 channel (690/50). Both fluorochromes were excited with a 488-nm laser and no spill compensation was applied. Two technical replicates of at least 10,000 sperm were analysed at constant flow rate, laser voltage and sperm concentration. The percentage of viable sperm corresponded to the SYBR-14+/PI- population, after subtracting the percentage of debris particles in the analysis.<br><br>Sperm intracellular calcium levels were assessed following Harrison, Mairet, & Miller, 1993; sperm were stained with Fluo3-AM (final concentration: 1.2 µM) and PI (final concentration: 5.6 µM) for 10 min at 38 ºC in the dark. Fluo3 was detected through the FITC channel (525/40). The mean of Fluo3 fluorescence intensity (Fluo3+/PI-) was recorded and used for statistical analysis.<br><br>Acrosome membrane integrity was evaluated following Nagy, Jansen, Toppe, & Gadella, 2003 protocol, in which sperm were stained with PNA-FITC (final concentration: 1.2 µM) for 5 min at 38 ºC in the dark, and then with PI (final concentration: 5.6 µM) for 5 min at 38 ºC in the dark. PNA-FITC was detected by the FITC channel (525/40). The percentage of viable sperm with an intact acrosome membrane (PNA-FITC-/PI-) was recorded and used for the subsequent statistical analysis<br><br>Mitochondrial membrane potential was evaluated following the protocol set by Ortega-Ferrusola et al., 2008. Sperm were incubated with JC-1 (final concentration: 750 nM) for 30 min at 38 ºC in the dark. In cells with high mitochondrial membrane potential, JC-1 aggregates and emits orange fluorescence, which is collected through the PE channel. On the contrary, in cells with low mitochondrial membrane potential, JC-1 is found in its monomeric form and generates green fluorescence, which is collected through the FITC channel. The percentage of sperm with high mitochondrial membrane potential was recorded and used for the subsequent statistical analysis.
Instrument	CytoFLEX cytometer
Software	CytExpert; Beckman Coulter Inc, 2.5.0.77; California, USA
Cell population abundance	No cell-sorting was used in the present work

Gating strategy

Forward (FS) and side scatter (SS) were measured and linearly recorded for all particles. Subcellular debris and cell aggregates were excluded, and sperm were positively gated, through the adjustment of the analyzer threshold on the FS channel. Finally, sperm-specific events were validated on the basis of FS/SS distributions.

☒ Tick this box to confirm that a figure exemplifying the gating strategy is provided in the Supplementary Information.
